# Mobile App–Assisted Parent Training Intervention for Behavioral Problems in Children With Autism Spectrum Disorder: Pilot Randomized Controlled Trial

**DOI:** 10.2196/52295

**Published:** 2024-10-28

**Authors:** JooHyun Lee, JaeHyun Lim, Soyeon Kang, Sujin Kim, So Yoon Jung, Sujin Kim, Soon-Beom Hong, Yu Rang Park

**Affiliations:** 1 Department of Biomedical Systems Informatics Yonsei University College of Medicine Seoul Republic of Korea; 2 LumanLab Inc Seoul Republic of Korea; 3 Division of Child and Adolescent Psychiatry Department of Psychiatry Seoul National University Hospital Seoul Republic of Korea; 4 Department of Psychiatry Seoul National University College of Medicine Seoul Republic of Korea; 5 Institute of Human Behavioral Medicine Seoul National University Medical Research Center Seoul Republic of Korea; 6 Department of Artificial Intelligence Yonsei University Seoul Republic of Korea

**Keywords:** autism spectrum disorder, parent training program, parent education, behavioral problems, child behavior, mobile app, feasibility, mHealth, evidence-based parent training

## Abstract

**Background:**

In children with autism spectrum disorder (ASD), problem behaviors play a dysfunctional role, causing as much difficulty with daily living and adjustment as the core symptoms. If such behaviors are not effectively addressed, they can result in physical, economic, and psychological issues not only for the individual but also for family members.

**Objective:**

We aimed to develop and evaluate the feasibility of a mobile app–assisted parent training program for reducing problem behaviors in children with ASD.

**Methods:**

This open-label, single-center, randomized controlled trial was conducted among parents of children with ASD aged 36-84 months. Participants were recruited from the Department of Psychiatry at Seoul National University Hospital. Participants were randomly assigned (1:1) by a blinded researcher. Randomization was performed using a stratified block randomization (with a block size of 4). Parents in the intervention group completed the mobile app–assisted parent training program at home over a 12-week period. They continued to receive their usual nondrug treatment in addition to the mobile app–assisted parent training program. The control group continued to receive their usual nonpharmaceutical treatment for 12 weeks without receiving the parent training program intervention. The primary outcome measure was the median change in the Korean Child Behavior Checklist (K-CBCL) scores from before to after the intervention. Lower scores on the K-CBCL indicated a decrease in overall problem behavior.

**Results:**

Between November 9, 2022, and December 8, 2022, 64 participants were enrolled. Overall, 42 children (intervention group median age: 49, IQR 41-52.5 months; control group median age: 49, IQR 42-58 months) of the participants joined the program. The intervention group included 20 (48%) participants and the control group included 22 (52%) participants. In the intervention group, the K-CBCL total scores showed a decrease after the intervention, with a median difference of –0.5 (95% CI –4.5 to 3). Pervasive developmental disorder scores also showed a decrease, with a median difference of –2.1 (95% CI –8.5 to 2.5). However, there was no significant difference in Clinical Global Impression–Severity of Illness scores after the intervention for both the control and intervention groups. Scores on the Korean version of the Social Communication Questionnaire showed a further decrease after the intervention in the intervention group (median difference –2, 95% CI –4 to 1). Caregivers’ stress evaluated using the Korean Parenting Stress Index Fourth Edition–Short Form did not show any significant differences between the control and intervention groups. There were no adverse events related to study participation.

**Conclusions:**

The findings demonstrated the feasibility of using mobile devices for evidence-based parent training to reduce problem behaviors in children with ASD. Mobile devices’ accessibility and flexibility may provide a viable alternative for offering early intervention for problem behaviors in children with ASD.

**Trial Registration:**

CRIS KCT0007841; https://cris.nih.go.kr/cris/search/detailSearch.do?&seq=23112

## Introduction

Autism spectrum disorder (ASD) is a condition that affects how a person interacts with others, communicates, and behaves. It is characterized by social interaction difficulties, communication impairments, repetitive behaviors, and limited attention [1]. The prevalence and economic cost of ASD have been gradually increasing in recent years in Korea and worldwide [2,3]. Facilities for psychological evaluation and treatment, including consultation with a pediatric psychiatrist and testing for ASD, are concentrated in metropolitan areas, making them inaccessible to children in rural areas [4,5]. Additionally, even when a diagnosis is made, treatment centers for speech therapy, psychotherapy, acceptance and commitment therapy, music therapy, and occupational therapy are equally concentrated in metropolitan areas; the high cost of treatment makes it difficult for low-income children to receive appropriate treatment [4,6]. Given its enduring consequences and significant socioeconomic burden, ASD is emerging as a priority for intervention efforts.

In individuals diagnosed with ASD, problem behaviors play a dysfunctional role, causing difficulties in daily living and adjustment as much as the core symptoms [7,8]. Problem behaviors can be defined as any behavior that is aggressive toward the self or others, causes damage to the physical environment or objects, interferes with acquiring new skills, or isolates the person from society [9]. Problem behaviors that are not effectively addressed can cause physical, economic, and psychological distress to primary caregivers and other family members [10,11]. Various nonpharmacologic treatments are available to address these problems in children with ASD. Among them, nondrug treatments such as acceptance and commitment therapy and music therapy have shown promising results in addressing emotional problems and improving overall well-being, but they are limited in their ability to directly target problem behaviors [12-16]. Applied Behavior Analysis (ABA) refers to evidence-based interventions that are applied to the education of children with ASD to increase desirable behaviors and decrease or eliminate undesirable behaviors [17,18]. ABA is the science of learning and behavior; to effectively intervene and change problem behaviors, it focuses on identifying the exact cause of the behavior and implementing interventions that target that cause [19,20]. ABA-based parent training is reportedly effective in reducing problem behaviors in children developing from preschool through adolescence in general. Parent training for children with ASD is practical as a treatment model, can be used in various settings, and empowers parents to be change agents themselves [21-23].

Digital therapeutics are advanced software technologies that deliver evidence-based therapeutic interventions to patients to prevent, manage, and treat diseases or disorders [24,25]. Korea’s high mobile phone use rate of 94.8% [26] highlights the potential for increasing accessibility to mobile app–assisted digital therapies. These therapies, with their scalability, accessibility, and low cost, offer the convenience of accessing treatment anytime, anywhere, making them particularly relevant for children in rural areas and low-income communities with limited access to hospitals and treatment centers [25,27].

This study aimed to (1) create a mobile app–assisted parent training program using digital technology to decrease problem behaviors in children with ASD, and (2) evaluate the effectiveness of the training program in reducing problem behaviors in children with ASD by comparing pre- and postintervention outcomes.

## Methods

### Study Design

**Figure 1:**
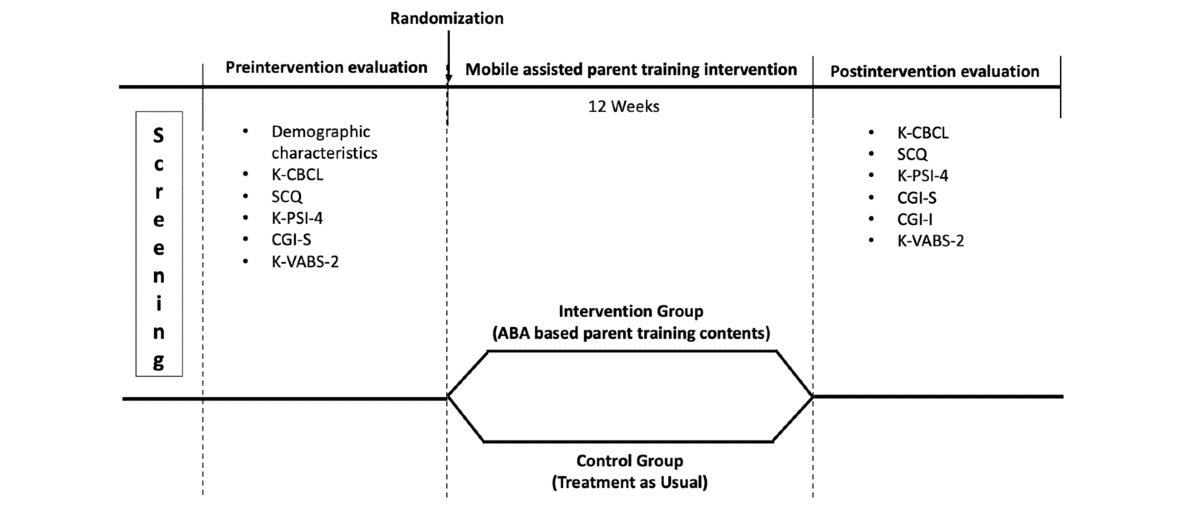
Study design. ABA: Applied Behavior Analysis; CGI-S: Clinical Global Impression–Severity of Illness; K-CBCL: Korean Child Behavior Checklist; K-PSI-4: Korean version of the Parenting Stress Index 4th Edition; K-VABS-2: Korean Vineland Adaptive Behavior Scale-2; SCQ: Social Communication Questionnaire.

This study was a parallel-group, open-label, single-center, randomized controlled trial to test the effectiveness of a mobile app–assisted parent training program for reducing behavior problems in children with ASD aged 36-84 months. It followed the CONSORT (Consolidated Standards of Reporting Trials) guidelines. A parent training program based on ABA [17,18] was delivered via mobile devices. For inclusion criteria, children of participants were recruited from the Department of Psychiatry at Seoul National University Hospital and underwent the Autism Diagnostic Observation Schedule-2 (ADOS-2) and Korean Childhood Autism Rating Scale-2 (K-CARS-2), provided demographic information, and completed before the evaluation. Parenting stress levels of the parents and caregivers were also measured. The eligible participants were randomly assigned to either the intervention group or the control group at a 1:1 ratio for 12 weeks. The intervention group received a mobile app parent training program for 12 weeks, while the control group continued with their usual treatment but received no intervention. Both groups were assessed before and after the 12-week period (Figure 1).

### Participants

Participants with eligible children were recruited from November 9, 2022, to December 8, 2023, at the Department of Psychiatry at Seoul National University Hospital. The inclusion criteria for the children were as follows: (1) being aged between 36 and 84 months of age; (2) meeting the criteria for “autism” or “autism spectrum” classification according to the ADOS-2; and (3) having as a primary caregiver an adult aged at least 19 years. The following were the exclusion criteria for this study: (1) the child was currently taking psychiatric medication for behavioral regulation or similar purposes; (2) the child had a history of congenital or acquired brain damage, such as cerebral palsy; (3) the child had a history of seizure disorders or other neurological conditions; (4) the child had an uncorrected sensory impairment (eg, vision or hearing deficits); (5) the child’s primary caregiver did not possess a smartphone or lacked access to smartphone apps; and (6) the primary caregiver either did not give their consent to join the study or opted to withdraw during the course of the study. All participants provided written informed consent.

### Randomization and Masking

The children were divided into low- and high-severity autism groups according to ADOS-2 criteria and randomized into intervention and control groups for each severity group. Randomization was performed using a stratified block randomization method (with a block size of 4) with 1:1 assignment to either the intervention or control group. The randomization list was created using the PROC PLAN method (SAS Institute Inc) in SAS (version 9.4; SAS Institute Inc) software. The randomization process was managed and operated by an independent third party, the Medical Statistics Team of Asan Medical Center.

### Sample Size

As this was an exploratory clinical trial, a formal calculation for sample size was not necessary; however, we followed the method described by Julious [28] to calculate the sample size for the pilot trial. The recommended sample size for such pilot studies is 12 persons per group. Considering the possibility of lower compliance with the use of digital therapeutics compared with taking conventional medicines, this study was conducted with a total of 60 participants, 30 in the intervention group and 30 in the control group.

### Mobile App–Assisted Parent Training Program

The mobile app–assisted parent training program aims to improve the behavior of children with ASD by identifying the causes of problem behaviors through a self-report questionnaire and providing digital content about it. Previous studies have shown that mobile app or telehealth-enabled ABA interventions can be effective in managing and reducing problem behaviors and increasing parent engagement in children with autism [29-31]. The program was developed in the form of a mobile app to help parents apply ABA-based therapy to their children in their daily lives to reduce their problem behaviors [32-34]. The mobile app–based training program lasted 10-15 minutes per session and was conducted 2-5 days a week for 12 weeks. It covers topics such as an introduction to ABA, identifying the underlying reasons for problematic behavior, creating a behavioral support plan, implementing behavior intervention, practicing in different situations, individualized learning opportunities, and crisis management (Table 1). To further validate our approach and differentiate it from previous studies, we included a system in the mobile app that allows parents to record and monitor their child’s behavior and progress. This real-time data collection allows for continuous monitoring and adjustment of the intervention to ensure responsiveness and effectiveness. Program content was developed with the consultation of 2 ABA experts and 1 child psychiatry specialist.

**Table 1:** Overview of the program curriculum of the mobile app–assisted parent training intervention for behavioral problems in children with autism spectrum disorder.

Session	Learning objectives	Description of the lesson
1	Orientation and introduction to ABA^a^	Orientation: Introducing good time ABA: definition, characteristics, and recent trends Defining objective behavior and collecting data
2	Identifying the reasons behind problematic behaviors	Functions of problem behaviors Methodology of functional assessment: indirect functional assessment, observational functional assessment (ABC^b^ functional assessment)
3	Creating a BSP^c^	Create a BSP according to ABC functional assessment: antecedent intervention; consequences intervention; behavior intervention
4	Antecedent intervention 1: Situation (background) event intervention	Finding and structuring environments that cause problematic behavior Finding and structuring time environments that cause problematic behavior
5	Antecedent intervention 2	Stimulus control Motivating operation
6	Consequences intervention 1: Reinforcement	Reinforcement and differential reinforcement Behavior contract and token reinforcement
7	Consequences intervention 2: Extinction	Extinction Disinterestedness
8	Behavior intervention 1: Instruction following and FCT^d^	Instruction following FCT
9	Behavior intervention 2: Behavior shaping and chaining	Behavior shaping Behavior chaining
10	Situation training	Introduce situation training Picture-supported situation training Word-supported situation training
11	DTT^e^	What is DTT? Procedure of DTT Application of DTT
12	Crisis management: Self-harm and aggressive behavior	Aggressive behavior Destructive behavior, self-stimulatory behavior, and self-harm Preparing a safety plan

^a^ABA: Applied Behavior Analysis.

^b^ABC: antecedents-behavior-consequences.

^c^BSP: behavior support plan.

^d^FCT: functional communication training.

^e^DTT: discrete trial training.

### Procedure

**Figure 2:**
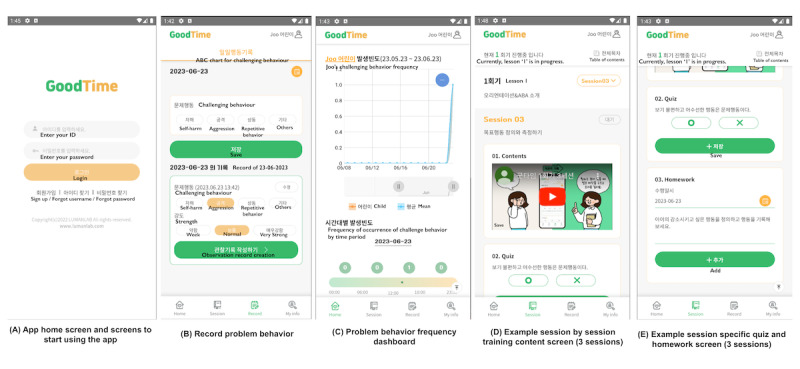
Mobile app platform example. (A) App home screen and screens to start using the app; (B) record problem behavior; (C) problem behavior frequency dashboard; (D) example session by session training content screen (3 sessions); and (E) example session-specific quiz and homework screen (3 sessions).

The mobile app–assisted parent training program was available for download from a mobile app store, and participants accessed this study’s intervention using a unique, preannounced username and password. Parents in the intervention group completed the mobile app parent training program at home over a 12-week period, during which they continued to receive their usual nondrug treatment in addition to the training program. The mobile app included recording problem behaviors and viewing the frequency of child problem behaviors, education, and homework assignments to help caregivers generalize skills in a real-world setting (Figure 2). All sessions included instruction on the parent training program, video content with case examples to help parents understand the program, and quizzes and homework to check for understanding of the session. Participants could access the app at any time, and all programs delivered were relearnable.

### Control Group

The control group continued to receive their usual nonpharmaceutical treatments, such as behavioral therapy, speech therapy, and play therapy, for 12 weeks, without receiving the mobile app–assisted parent training program intervention.

### Measures

#### About ADOS-2

The ADOS-2 is a tool used to help diagnose ASD by observing children’s social interactions and behaviors through play or conversation [35]. It is administered by a qualified evaluator and takes approximately 45 minutes to complete. It is used to set standards for participating children and is a semistructured tool for directly observing children [36].

#### About K-CARS-2

The K-CARS-2 is a tool used to differentiate between autism and other developmental disorders, and to determine the severity of autism disorders [37,38]. It can be completed in a relatively short amount of time, typically taking 20-30 minutes.

#### Korean Child Behavior Checklist

The Korean Child Behavior Checklist (K-CBCL) is an evaluation tool used to assess the impact of overall problem behaviors, adaptation, and social performance in children [39]. It is a standardized checklist completed by parents, who describe their child’s behavioral and emotional problems. The K-CBCL comprises a social ability scale and a syndrome and total problem scale, taking 15-20 minutes to complete. It is suitable for infants and toddlers aged 18 months to 5 years and can be administered to children aged up to 6 years in kindergarten.

#### About K-SCQ

The Korean Version of the Social Communication Questionnaire (K-SCQ) is a useful screening tool for identifying a range of symptoms related to ASD in a short amount of time (approximately 15 min) and can be used with people of any age and language level. It comprises 2 types of tools, each containing 40 questions that ask parents or guardians about their child’s symptoms related to autism (such as communication, interaction, and limited and repetitive behaviors and interests) [40]. The Social Communication Questionnaire (SCQ) current type focuses on behavior over the past 3 months, while the SCQ lifetime type focuses on behavior over a lifetime. This study used the Korean translation of the SCQ current type [41].

#### About Korean Vineland Adaptive Behavior Scale-2

The Korean Vineland Adaptive Behavior Scale-2 (K-VABS-2) is an evaluation tool used to assess the impact of overall problem behaviors, adaptation, and social performance in individuals of all ages. It comprises 4 areas (communication, daily life technology, socialization, and exercise functions) and 11 subareas (including language skills, coping abilities, and small muscle control). The K-VABS-2 can be administered in the form of a survey interview, taking 20-60 minutes, or a guardian rating, taking 30-60 minutes. It is suitable for individuals of all ages [42].

#### Clinical Global Impression–Severity of Illness

In the Clinical Global Impression–Severity of Illness (CGI-S), a physician uses a scale of 1-7 points to evaluate the severity of a disease based on the symptoms experienced by past patients diagnosed with the same disease. This scale is used to determine the degree of symptoms in currently diagnosed patients [43].

#### Clinical Global Impression Global Improvement

In the Clinical Global Impression Global Improvement (CGI-I), a physician uses a scale of 1-7 points to evaluate the effectiveness of therapeutic intervention in a patient with a mental disorder. The scale is used to determine whether the patient’s condition has improved or worsened compared with before the intervention was initiated [43].

#### Korean Parenting Stress Index Fourth Edition–Short Form

The Parenting Stress Index is a self-report test used to measure the stress experienced by parents of children aged 1-12 years. It evaluates the characteristics and situational factors of children that affect parenting stress as perceived by the parents [44] and comprises 2 main areas: child and parent. The child area is further divided into 6 subscales: distraction or excessive behavior, adaptation, compensation, demand, mood, and acceptance. The parent area comprises 7 subscales: competence, isolation, attachment, health, role restriction, depression, and spouse or parenting partner relationship. The test also includes a life stress scale that measures events that can affect parenting stress. The Parenting Stress Index is available in both a general form and a short form (Korean Parenting Stress Index Fourth Edition–Short Form [K-PSI-4-SF]); the latter was used in our study [45].

### Data Management

Research data were stored in a secure laboratory and personal information was stored in a separate file to ensure that personal identification is not possible through the research data. Enrollment logs were used to maintain personal identification information separately; the case report form did not include personal information except for initials and case numbers. This helped to protect participants’ privacy.

### Statistical Analysis

All outcomes were analyzed in participants who completed the program period and assessments at baseline and after the intervention. The analysis included participants who did not violate the protocol (per-protocol analysis set). Owing to the nonnormal distribution, nonparametric methods were used in this study. All baseline demographic variables and evaluation outcomes at baseline and after the intervention were summarized by randomized groups using the median (IQR) for continuous data or count (%) for categorical data. We estimated the median values and IQRs for each group and time point using the pre- and postintervention evaluation scores for the intervention and control groups. Before testing the effectiveness of the program, the Wilcoxon signed rank test was performed to determine whether there were differences in demographic and baseline variables between the groups. The Wilcoxon signed rank test was used to compare the effects of all outcomes. To evaluate the intervention effects between the 2 groups, the Kruskal-Wallis test was performed by judging the rejection range based on a significance level of 0.05. To assess differences in session completion rates within the intervention group, we estimated medians and IQRs and used Wilcoxon signed rank tests. To examine the correlation of completion rates with evaluation outcomes, Pearson correlation coefficient was used. Data analysis was performed using the R software (version 4.1.0; R Foundation) and Python (version 3.9.12; Python Software Foundation).

### Ethical Considerations

Written informed consent was obtained from all participants. This study was approved by the Seoul National University Hospital Institutional Review Board (H-2205-158-1329).

All data were stored in a locked laboratory, and participants’ personal information was stored in a separate file from the research data to prevent identification through the research data. Enrollment logs were maintained to keep personal information separate and to ensure that personal information was not exposed. Only initials and case numbers were used on case report forms to further protect participant identity.

The tests administered as part of this study were provided free of charge. Compensation was not contingent on the participant completing this study, and pretest results were provided even if the participant did not complete this study. The posttest was only administered if the participant completed this study. No other monetary compensation, including transportation, was offered.

## Results

### Participant Characteristics

**Figure 3:**
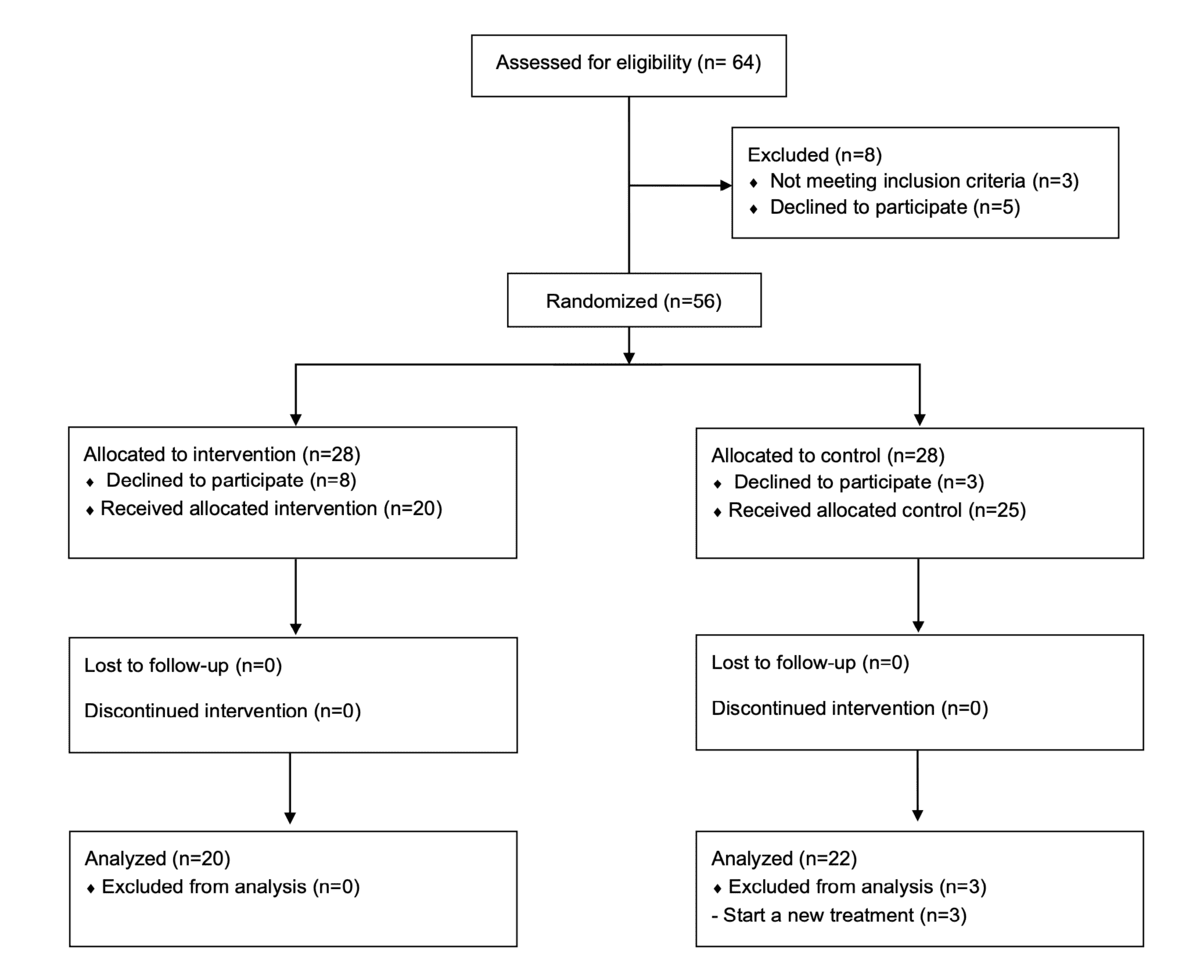
CONSORT flowchart of participation. CONSORT: Consolidated Standards of Reporting Trials.

Between November 9, 2022, and December 8, 2022, a total of 64 participants were enrolled. Further, 56 participants who met our inclusion criteria were randomly assigned into the intervention and control groups; however, after randomization, 8 participants in the intervention group and 3 in the control group declined to participate, resulting in 20 participants receiving the intervention and 25 participants in the control group. In the control group, 3 participants were excluded from the analysis; these included children who started a new treatment during the intervention period. Finally, a total of 42 participants were included in the data analysis (Figure 3).

Demographic and baseline variables were compared between the intervention and control groups before the intervention. Of the 42 participants, 20 (48%) were in the intervention group and 22 (52%) in the control group. There were no significant differences in sex, age, severity, or ethnicity between the 2 groups. Demographic variables, including the K-CARS-2 and ADOS-2, did not differ significantly between the groups. The current treatment also did not differ significantly between the intervention (n=17, 85%) and control (n=18, 81.8%) groups. Neuropsychological testing showed no significant differences between the 2 groups. The demographic and baseline variables of both groups are summarized in Table 2. Complete information is presented in Table S1 in Multimedia Appendix 1. Table S2 in Multimedia Appendix 1 provides demographic and baseline information for the full sample using an intention-to-treat (ITT) analysis approach. The results of this ITT analysis revealed a significant difference between the intervention group (14.5, 95% CI 12.5 to 16) and the control group (12, 95% CI 10 to 15) on social effect, a subscale of the ADOS, compared with the results obtained from the per-protocol analysis (*P*=.045). Except for this item, no significant differences were found for the remaining items. Multimedia Appendix 1 provides more details.

**Table 2:** Demographic and baseline variables for the intervention and control groups (a version of per-protocol analysis).

Characteristics				Intervention group (N=20)		Control group (N=22)
**Sex** **, n (%)**						
	Male		15 (75)		18 (81.8)	
	Female		5 (25)		4 (18.2)	
Age (months), median (IQR)				49 (41 to 52.5)		49 (42 to 58)
**Severity, n (%)**						
	Mild		4 (20)		4 (18.2)	
	Severe		16 (80)		18 (81.8)	
K-CARS-2^a^, median (IQR)				33 (32-36.2)		35 (31-37)
**ADOS^b^** **comparison, n (%)**						
	Extreme		11 (55)		11 (50)	
	Moderate		1 (5)		3 (13.6)	
	Moderate to severe		2 (10)		1 (4.5)	
	Severe		6 (30)		7 (31.8)	
**Ongoing therapy, n (%)**						
		No	3 (15)		4 (18.2)	
		Yes	17 (85)		18 (81.8)	
**Physician measures**						
	CGI-S^c^, median (IQR)		3 (2 to 4)		3 (2 to 4)	
**Parents’ measures (for children), median (IQR)**						
	**K-CBCL^d^** **(total)**		57.5 (50 to 63)		60.5 (55 to 64)	
		Emotionally reactive	50 (50 to 58)		53 (50-63)	
		Anxious or depressed	50 (50 to 58)		50 (50-56)	
		Somatic concerns	54 (50 to 59)		50 (50-54)	
		Withdrawn	66.5 (62 to 70)		62 (58-65)	
		Attention problems	61.5 (55 to 66)		59 (50-64)	
		Aggressive behavior	53.5 (50 to 57.5)		57 (50-64)	
	K-SCQ^e^ (total)		18 (13 to 20)		16 (12 to 22)	
	**K-VABS-2^f^**					
		Communication	64 (57 to 70)		66 (53 to 81)	
		Daily living skills	66 (59 to 69)		66 (59 to 80)	
		Socialization	51 (46.5 to 54)		50 (48 to 60)	
		Motor	67 (61 to 80)		68.5 (58 to 80)	
		Maladaptation	18 (17 to 20)		19.5 (19 to 20)	
**Parents’ measures (for parents), median (IQR)**						
	**K-PSI-4-SF^g^**					
		Total stress scale	103 (93 to 113)		95 (88 to 109)	
		Parental distress	34 (31.5 to 38.5)		33.5 (28 to 40)	
		Parent-child dysfunctional interaction	34 (30.5 to 38)		29 (26 to 38)	
		Difficult child	32.5 (29.5 to 38.5)		30 (26 to 37)	

^a^K-CARS-2: Korean Childhood Autism Rating Scale-2.

^b^ADOS: Autism Diagnostic Observation Schedule.

^c^CGI-S: Clinical Global Impression–Severity of Illness.

^d^K-CBCL: Korean Child Behavior Checklist.

^e^K-SCQ: Korean versions of the Social Communication Questionnaire.

^f^K-VABS-2: Korean Vineland Adaptive Behavior Scale-2.

^g^K-PSI-4-SF: Korean Parenting Stress Index 4th Edition–Short Form.

### Primary Outcome

The results for the K-CBCL scores are shown in Table 3. According to the K-CBCL, lower scores indicate a decrease in overall problem behaviors. The K-CBCL total score for the intervention group decreased from baseline to after the intervention (median difference –0.5, 95% CI –4.5 to 3). In comparison to the control group, the intervention group’s median withdrawn score difference reduced to –2 (95% CI –8 to 4), median difference of attention decreased by –1 (95% CI –6 to 4), and median difference in pervasive developmental disorder exhibited a –1 (95% CI –5 to 2) decrease. The full table is shown in Table S3 in Multimedia Appendix 1.

**Table 3:** Comparison between outcome variables for the intervention and control groups (a version of per-protocol analysis).

Characteristics				Intervention									Control									Median difference, (95% CI)			*P* value
				Pre (n=20), median (IQR)		Post (n=20), median (IQR)		Median difference, (95% CI)		*P* value		Pre (n=22), median (IQR)			Post (n=22), median (IQR)		Median difference, (95% CI)		*P* value						
**Primary outcomes**																									
	**K-CBCL^a^ (total)^b^**			57.5 (50 to 63)		58 (50 to 63)		–0.5 (–4.5 to 3)		.91		60.5 (55 to 64)			55.5 (52 to 65)		–2 (–6 to 2)		.43		2 (–4 to 6)			.50	
		Withdrawn	66.5 (62 to 70)		68 (60 to 71.5)		1 (–4.5 to 6.5)		.78		62 (58 to 65)			68 (62 to 70)		4 (–2.5 to 8)		.23		–2 (–8 to 4)			.50		
		Attention problems	61.5 (55 to 66)		61.5 (55 to 66)		0 (–4.5 to 6.5)		.95		59 (50 to 64)			61.5 (55 to 68)		1.5 (–2 to 7)		.40		–1 (–6 to 4)			.55		
		Pervasive developmental disorder	69 (64 to 74)		69.5 (59.5 to 72)		–2.1 (–8.5 to 2.5)		.41		69.5 (66 to 76)			69.5 (66 to 76)		1 (–4.5 to 4)		.74		–1 (–5 to 2)			.41		
**Physician measures**																									
	CGI–S^c^			3 (2 to 4)		3 (2 to 3)		–0.5 (–2 to 1)		.40		3 (2 to 4)			3 (2 to 3)		–1 (–2 to 1)		.23		0 (0 to 0.5)			.70	
**Secondary outcomes**																									
	K–SCQ^d^ (total)^b^			18 (13 to 20)		15 (9.5 to 20)		–2 (–4 to 1)		.20		16 (12 to 22)			16.5 (9 to 20)		–1.5 (–3 to 0)		.08		0 (–3 to 3)			.90	
	**K–VABS–2^e,f^**																								
		Communication	64 (57 to 70)		68 (56 to 75)		4 (0 to 8)		.04		66 (53 to 81)			73 (52 to 85)		0 (–5 to 3)		.84		4 (0 to 10)			.09		
		Daily living skills	66 (59 to 69)		69 (63 to 74)		4.5 (–2.5 to 14.5)		.12		66 (59 to 80)			66 (49 to 76)		–5.1 (–9.5 to 0.5)		.06		6 (1 to 13)			.02		
		Socialization	51 (46.5 to 54)		55 (48 to 62.5)		7 (2.5 to 12)		<.01		50 (48 to 60)			51 (48 to 56)		–1.5 (–5.5 to 2)		.55		7 (2 to 13)			<.01		
**Parents’ measures (for parents)**																									
	**K–PSI–4–SF^b,g^**																								
		Total stress scale	103 (93 to 113)		106.5 (93 to 116)		0.5 (–4.5 to 6.5)		.88		95 (88 to 109)			93.5 (87 to 108)		–3.5 (–9 to 3)		.20		4 (–3 to 11)			.19		
		Parental distress	34 (31.5 to 38.5)		37 (32 to 41.5)		1.5 (–1.5 to 5)		.29		33.5 (28 to 40)			32.5 (28 to 38)		–1.5 (–3.5 to 0.5)		.15		2 (0 to 6)			.07		

^s^K-CBCL: Korean Child Behavior Checklist.

^b^Lower score indicates increased ability.

^c^CGI-S: Clinical Global Impression–Severity of Illness.

^d^K-SCQ: Korean versions of the Social Communication Questionnaire.

^e^K-VABS-2: Korean Vineland Adaptive Behavior scale-2.

^f^Higher score indicates increased ability.

^g^K-PSI-4-SF: Korean Parenting Stress Index 4th Edition–Short Form.

### Secondary Outcomes

#### Overview

A lower CGI-S score indicates less severe symptoms, a lower K-SCQ score indicates better social communication ability, a lower K-PSI-4-SF score indicates less stress, and a higher K-VABS-2 score indicates better adaptation and social performance. The results for the secondary outcomes are shown in Table 3. Table S3 and Figure S1 in Multimedia Appendix 1 show the full table and plots.

#### Clinical Global Impression

There was no median difference between baseline and after the intervention for the intervention and control groups on the CGI-S (0, 95% CI 0 to 0.5). On the CGI-I, the number of participants who improved after the intervention was 17 (77.3%) in the control group and 17 (85%) in the intervention group.

#### Social Adoption and Emotional and Behavioral Problems

K-SCQ scores in the intervention group decreased further after the intervention (median 15, IQR 9.5-20) compared with baseline (median 18, IQR 13-20). The median difference in communication on the K-VABS-2 exhibited a 4 (95% CI 0 to 8) increase after the intervention compared with the baseline, and socialization (7, 95% CI 2 to 13; *P* value <.01) increased more in the intervention group compared with the control group. Daily living skills also showed a significant difference when comparing the pre-post difference in the intervention group to the pre-post difference in the control group (6, 95% CI 1 to 13; *P* value .02).

#### Caregiver’s Stress

Caregiver’s stress was evaluated using the K-PSI-4-SF. The median difference between the intervention and control groups showed that the total stress scale (4, 95% CI –3 to 11), parental distress (2, 95% CI 0 to 6), parent-child dysfunctional interaction (1, 95% CI –2 to 4), and difficult child (0.5, 95% CI –3 to 3) were not statistically different between the intervention and control groups.

#### Comparison Values Based on Session Completion Rate

The mean number of sessions completed by participants in the intervention group was 13.4 of 26 (51.5%) sessions. The median pre- and post difference for K-CBCL total was –3.5 (95% CI –12 to 5) for 100% completion and –3.5 (95% CI –8.5 to 3) for 40% completion. The median difference in withdrawn was –4.5 (95% CI –14 to 5) for 100% completion and –3.1 (95% CI –11 to 6) for 40% completion, indicating a decrease in the intervention effect. For sleep problems, the median difference was –12 (95% CI not available) for 100% completion and –8.3 (95% CI –12 to –1.5) for 40% completion, indicating a decrease in the intervention effect. Communication and motor skills in the K-VABS-2 also increased from 8 (95% CI –8 to 24) and 10.8 (95% CI 2 to 22) at 100% completion to 3 (95% CI –4 to 11) and 5.5 (95% CI –0.5 to 17.5) at 40% completion, respectively. The total stress scale on the K-PSI-4-SF was 9.5 (95% CI 1.5 to 17) at 100% completion and 1.5 (95% CI –6.5 to 12) at 40% completion, indicating that higher session completion rates were associated with higher caregiver stress. All results for pre- and post median differences in evaluation scores by session completion rate are shown in Table 4 and Table S5 in Multimedia Appendix 1, and correlations for pre- and post median differences in evaluation score by session completion rate are shown in Figure S2 in Multimedia Appendix 1.

**Table 4:** Differences in pre- and postevaluation values by session completion rate.

Characteristics				Session completion rate							
				100%^a^ (n=5), median difference, median (95% CI)		90%^b^ (n=6), median difference, median (95% CI)	80%^c^ (n=7), median difference, median (95% CI)		60%^d^ (n=8), median difference, median (95% CI)		40%^e^ (n=9), median difference, median (95% CI)
**Primary outcomes**											
	**K-CBCL (total)^f,g^**			–3.5 (–12 to 5)		–3 (–8.5 to 4.5)	–3.4 (–8.5 to 4.5)		–3.5 (–8.5 to 4.5)		–3.5 (–8.5 to 3)
		Anxious or depressed	5.5 (5.5 to 5.5)		6.8 (–2 to 13)		6.8 (–2 to 13)	6.8 (–2 to 13)		3.1 (–6 to 13)	
		Withdrawn	–4.5 (–14 to 5)		–2 (–8 to 5)		–2 (–8 to 5)	–3.5 (–10.5 to 5)		–3.1 (–11 to 6)	
		Sleep problems	–12 (N/A^h^ to N/A)		–8.5 (–8.5 to –8.5)		–9.1 (–12 to –12)	–9.1 (–12 to –12)		–8.3 (–12 to –1.5)	
		Aggressive behavior	–1 (–1 to –1)		–1 (–5 to 3)		–0.4 (–5 to 3)	0.5 (–5 to 3)		–0.5 (–3 to 2.5)	
**Physician measures**											
	CGI-S^g,i^			–0.5 (–0.5 to –0.5)		–0.2 (1 to 1)	–0.4 (–0.5 to 1)		–0.4 (–0.5 to 1)		–0.4 (–0.5 to 1)
**Secondary outcomes**											
	K-SCQ^j^ (total)^g^			–5.5 (–7 to –4)		–5 (–6 to –1)	–4.5 (–6 to –0.5)		–4 (–6.5 to –0.5)		–3 (–6 to 1)
	**K-VABS-2^k,l^**										
		Communication	8 (–8 to 24)		8 (–2 to 16)		3.8 (–6 to 17)	3 (–5 to 16)		3 (–4 to 11)	
		Motor	10.8 (2 to 22)		10.8 (2 to 22)		8 (2 to 22)	5 (0 to 13)		5.5 (–0.5 to 17.5)	
**Parents’ measures (for parents)**											
	**K-PSI-4-SF^g,m^**										
		Total stress scale	9.5 (1.5 to 17)		6.5 (–4 to 17)		4.1 (–8.5 to 20)	2.4 (–7.5 to 15)		1.5 (–6.5 to 12)	
		Parental distress	5.5 (–2 to 14)		5 (–1.5 to 9.5)		1.5 (–7 to 13)	2 (–6.5 to 10)		2.5 (–3 to 9.5)	
		Parent-child dysfunctional interaction	1.3 (–6 to 3)		–1.5 (–6 to 2)		–1.5 (–4 to 2)	–2 (–5.5 to 2)		–1.7 (–5.5 to 2)	
		Difficult child	4.6 (–2 to 10)		4 (0.5 to 8)		3 (0.5 to 8)	2.9 (–5 to 9)		1.3 (–5 to 6.5)	

^a^Groups with a session completion rate of 100% for the program.

^b^Groups with a session completion rate of 90% or higher for the program.

^c^Groups with a session completion rate of 80% or higher for the program.

^d^Groups with a session completion rate of 60% or higher for the program.

^e^Groups with a session completion rate of 40% or higher for the program.

^f^K-CBCL: Korean Child Behavior Checklist.

^g^Lower score indicates increased ability.

^h^N/A: not available.

^i^CGI-S: clinical global impression–severity of illness.

^j^K-SCQ: Korean versions of the Social Communication Questionnaire.

^k^K-VABS-2: Korean Vineland adaptive behavior scale-2

^l^Higher score indicates increased ability.

^m^K-PSI-4-SF: Korean Parenting Stress Index 4th Edition–Short Form.

### Statement on Harm

There were no serious intervention-related adverse events that led to treatment discontinuation in either group (Table 5).

**Table 5:** Summary of any adverse events during the trial.

Adverse events	Intervention group (n=20), n (%)	Control group (n=22), n (%)
Yes	0 (0)	0 (0)
No	20 (100)	22 (100)

## Discussion

### Principal Findings

This study tested the feasibility of a mobile app–assisted parent training program by comparing pre- and postintervention outcomes over a 12-week intervention to evaluate the effectiveness of the program in reducing behavioral problems in children with autism. Specifically, this study demonstrated the potential for a mobile app–based intervention to reduce behavioral problems in children with ASD. These findings can help in the planning of early interventions and high accessibility of mobile app–assisted parent training programs for children with ASD who have behavioral issues. Problem behaviors in children with ASD cause additional challenges for parents and create uncertainty about how to manage these behaviors. Given these challenges, parents of children with ASD can handle behavioral issues through parent training [46].

Regarding this study’s primary outcome, we found no clinically important differences between the intervention and control groups. A possible explanation for these results is that both groups maintained their existing treatments, such as ABA therapy, sensory integration therapy, and language therapy, which may have provided an indirect pathway for improving problem behaviors in children with ASD. However, the results can still be considered clinically significant. The pre- and posttreatment K-VABS-2 scores for the intervention group showed clinically significant effects for both communication and socialization. Daily living skills also showed clinically significant effects when comparing the intervention and control groups (Table 2). These scores represent the impact of overall problem behaviors, adaptation, and social performance in children, with similar results to previous studies [47,48]. Considering the exploratory nature of this study, we used the K-CBCL as the primary outcome measure, which provides subscale scores across various measurements. However, the actual significant effects were observed in core areas wherein individuals with ASD face difficulties, such as socialization, as was evident in the K-VABS-2, a widely used measure of adaptive behaviors in children with developmental disorders. These findings suggest the potential for conducting a confirmatory study using more focused outcome measurements.

Results on parenting stress showed that, within the intervention group, caregivers who used the training program the most reported higher levels of parenting stress (Table 3 and Figure S2 in Multimedia Appendix 1). This may imply that the more parenting stress a caregiver has, the more extensively they use the program.

Analyzing the results based on session completion rates, a more pronounced difference was observed between K-CBCL and K-VABS-2. While the scores measured by K-CBCL did not exhibit significant variations based on different completion rates, a notable difference was found in the K-VABS-2 scores. One of the key advantages of digital interventions is their relative freedom from constraints such as time, space, and cost. Therefore, a higher session completion rate would have been expected. However, considering that the session completion rate in this study was not as high as anticipated, our data suggest the need for complementary strategies to enhance user engagement. In doing so, if parental participation in this training program increases, our study’s findings indicate the potential for further improvements in the K-VABS-2 scores.

Ongoing research suggests that a significant number of parents are interested in mobile app–based, evidence-based parent training programs, which do not rely on therapists because the content is standardized and can be easily accessed by parents anytime and anywhere [49]. Using these mobile app technologies can help bring key elements of evidence-based interventions to populations that may not otherwise be able to receive them.

This study had several limitations. First, it relied on parental opinions to assess children’s behavior. Although the CGI-S and CGI-I were assessed by a physician, as the physician’s evaluation relied on parental reports regarding the child, it was also influenced by the parents’ opinions. Second, we observed a nonsignificant clinical effect of the treatment group on the primary outcome, which we attribute to indirect effects from other treatments the child was already receiving owing to this study’s nature. Third, our study had low program participation rates. For studies based on mobile app interventions, there are challenges in promoting participant engagement in the program.

### Conclusions

This study demonstrated the feasibility of using mobile devices for evidence-based parent training to reduce problem behaviors in children with ASD. In addition to the high prevalence of ASD and the high cost of treatment, there is a significant shortage of people to provide treatment, which is a barrier to treatment delivery. The accessibility and flexibility of mobile devices may make them a viable alternative as a parent education tool in providing early intervention for problem behaviors in children with ASD.

## Appendix

Multimedia Appendix 1 Tables and figures for intervention and control groups and pre and post evaluation values.

Multimedia Appendix 2 CONSORT-eHEALTH checklist (V 1.6.1).

## Abbreviations

ABA: Applied Behavior Analysis
ADOS-2: Autism Diagnostic Observation Schedule-2
ASD: autism spectrum disorder
CGI-I: Clinical Global Impression Global Improvement
CGI-S: Clinical Global Impression–Severity of Illness
CONSORT: Consolidated Standards of Reporting Trials
ITT: intention-to-treat
K-CARS-2: Korean Childhood Autism Rating Scale-2
K-CBCL: Korean Child Behavior Checklist
K-PSI-4-SF: Korean Parenting Stress Index Fourth Edition–Short Form
K-SCQ: Korean version of the Social Communication Questionnaire
K-VABS-2: Korean Vineland Adaptive Behavior Scale-2
SCQ: Social Communication Questionnaire

## Data Availability

The datasets produced or examined during this study are not openly accessible owing to the sensitivity of the information they contain and the potential risk of identifying individuals involved. Nonetheless, researchers who meet the necessary qualifications may be able to access these data following a structured request procedure that necessitates ethical clearance. Inquiries for data access should be directed to the corresponding author and will be evaluated individually.
