# Supplementary material for: Mobile App–Assisted Parent Training Intervention for Behavioral Problems in Children With Autism Spectrum Disorder: Pilot Randomized Controlled Trial
Source: JMIR Hum Factors. 2024 Oct 28;11:e52295. doi: 10.2196/52295 (PMC11555457; doi:10.2196/52295)
Supplement: Multimedia Appendix 1 [file humanfactors_v11i1e52295_app1.docx]

**Table of Contents**

**Table S1:** Demographic and baseline variable for intervention and Control Group (a version of Per Protocol)

**Table S2:** Demographic and baseline variable for intervention and Control Group (a version of Intention-to-treat)

**Table S3.** Comparison between outcome variables for the intervention and control groups (a version of Per Protocol)

**Table S4.** Comparison between outcome variables for the intervention and control groups (a version of Intention-to-treat)

**Table S5.** Difference in Pre and Post evaluation Values by Session Completion Rate

**Figure S1.** Comparison between CGI-I for the intervention and Control groups

**Figure S2.** Correlate differences in pre- and post-intervention evaluation results by session completion rate

**Table S1.** Demographic and baseline variable for intervention and control groups (a version of per protocol).

|  | Group | | *P* |
| --- | --- | --- | --- |
| Characteristics | Intervention  (N=20) | Control  (N=22) |  |
| Sex, n (%) |  |  | 0.872 |
| - Male | 15 (75.0%) | 18 (81.8%) |  |
| - Female | 5 (25.0%) | 4 (18.2%) |  |
| Age (Months), median (IQR) | 49.0 (41.0 to 52.5) | 49.0 (42.0 to 58.0) | 0.605 |
| Severity, n (%) |  |  | 1.000 |
| - Mild | 4 (20.0%) | 4 (18.2%) |  |
| - Severe | 16 (80.0%) | 18 (81.8%) |  |
| Ethnicity, n (%) |  |  | 1.000 |
| - Asian | 20 (100.0%) | 22 (100.0%) |  |
| K-CARS-2, median (IQR) | 33.0 (32.0 to 36.2) | 35.0 (31.0 to 37.0) | 0.860 |
| ADOS module, n (%) |  |  | 0.385 |
| - Module 1 | 16 (80.0%) | 16 (72.7%) |  |
| - Module 2 | 3 (15.0%) | 6 (27.3%) |  |
| - Toddle | 1 (5.0%) | 0 (0.0%) |  |
| ADOS (Total), median (IQR) | 18.5 (17.0 to 20.0) | 16.0 (14.0 to 19.0) | 0.117 |
| - Social affect | 14.5 (12.5 to 16.0) | 12.5 (10.0 to 15.0) | 0.104 |
| - Repetitive behaviors | 4.0 (3.0 to 5.0) | 4.0 (2.0 to 5.0) | 0.635 |
| ADOS Comparison, n (%) |  |  | 0.725 |
| - Extreme | 11 (55.0%) | 11 (50.0%) |  |
| - Moderate | 1 (5.0%) | 3 (13.6%) |  |
| - Moderate to Severe | 2 (10.0%) | 1 (4.5%) |  |
| - Severe | 6 (30.0%) | 7 (31.8%) |  |
| **Current Therapies, n (%)** |  |  |  |
| Ongoing intervention |  |  | 1.000 |
| - No | 3 (15.0%) | 4 (18.2%) |  |
| - Yes | 17 (85.0%) | 18 (81.8%) |  |
| ABA |  |  | 1.000 |
| - No | 14 (70.0%) | 16 (72.7%) |  |
| - Yes | 6 (30.0%) | 6 (27.3%) |  |
| Sensory integration therapy |  |  | 0.178 |
| - No | 5 (25.0%) | 11 (50.0%) |  |
| - Yes | 15 (75.0%) | 11 (50.0%) |  |
| Language therapy |  |  | 0.849 |
| - No | 4 (20.0%) | 6 (27.3%) |  |
| - Yes | 16 (80.0%) | 16 (72.7%) |  |
| Music therapy |  |  | 1.000 |
| - No | 20 (100.0%) | 21 (95.5%) |  |
| - Yes | 0 (0.0%) | 1 (4.5%) |  |
| Play therapy |  |  | 1.000 |
| - No | 12 (60.0%) | 13 (59.1%) |  |
| - Yes | 8 (40.0%) | 9 (40.9%) |  |
| Floortime |  |  | 0.512 |
| - No | 20 (100.0%) | 20 (90.9%) |  |
| - Yes | 0 (0.0%) | 2 (9.1%) |  |
| Cognition therapy |  |  | 1.000 |
| - No | 19 (95.0%) | 21 (95.5%) |  |
| - Yes | 1 (5.0%) | 1 (4.5%) |  |
| Occupational therapy |  |  | 1.000 |
| - No | 18 (90.0%) | 19 (86.4%) |  |
| - Yes | 2 (10.0%) | 3 (13.6%) |  |
| Art therapy |  |  | 0.962 |
| - No | 19 (95.0%) | 22 (100.0%) |  |
| - Yes | 1 (5.0%) | 0 (0.0%) |  |
| Developmental therapy |  |  | 1.000 |
| - No | 19 (95.0%) | 20 (90.9%) |  |
| - Yes | 1 (5.0%) | 2 (9.1%) |  |
| Social Skills therapy |  |  | 1.000 |
| - No | 19 (95.0%) | 20 (90.9%) |  |
| - Yes | 1 (5.0%) | 2 (9.1%) |  |
| Group therapy |  |  | 0.512 |
| - No | 20 (100.0%) | 20 (90.9%) |  |
| - Yes | 0 (0.0%) | 2 (9.1%) |  |
| **Physician measures** |  |  |  |
| CGI-S, median (IQR) | 3.0 (2.0 to 4.0) | 3.0 (2.0 to 4.0) | 0.887 |
| **Parents’ measures (for children)**, median (IQR) |  |  |  |
| K-CBCL (Total) | 57.5 (50.0 to 63.0) | 60.5 (55.0 to 64.0) | 0.588 |
| - Emotionally reactive | 50.0 (50.0 to 58.0) | 53.0 (50.0 to 63.0) | 0.115 |
| - Anxious/Depressed | 50.0 (50.0 to 58.0) | 50.0 (50.0 to 56.0) | 0.943 |
| - Somatic complaints | 54.0 (50.0 to 59.0) | 50.0 (50.0 to 54.0) | 0.414 |
| - Withdrawn | 66.5 (62.0 to 70.0) | 62.0 (58.0 to 65.0) | 0.118 |
| - Sleep problem | 50.0 (50.0 to 60.5) | 55.0 (50.0 to 62.0) | 0.418 |
| - Attention problems | 61.5 (55.0 to 66.0) | 59.0 (50.0 to 64.0) | 0.376 |
| - Aggressive behavior | 53.5 (50.0 to 57.5) | 57.0 (50.0 to 64.0) | 0.197 |
| - Other | 59.0 (55.0 to 64.5) | 64.0 (54.0 to 67.0) | 0.447 |
| - Internalizing domain | 56.5 (49.0 to 61.0) | 56.0 (54.0 to 60.0) | 0.990 |
| - Externalizing domain | 55.0 (49.0 to 63.0) | 58.5 (51.0 to 62.0) | 0.504 |
| - Affective disorder | 50.0 (50.0 to 58.0) | 50.0 (50.0 to 62.0) | 0.619 |
| - Anxiety disorder | 53.0 (50.0 to 59.0) | 53.0 (50.0 to 59.0) | 0.885 |
| - Pervasive developmental disorder | 69.0 (64.0 to 74.0) | 69.5 (66.0 to 76.0) | 0.535 |
| - Attention-Deficit/Hyperactivity disorder | 56.0 (52.0 to 64.5) | 59.0 (56.0 to 59.0) | 0.683 |
| - Oppositional defiant disorder | 53.5 (50.0 to 60.0) | 56.0 (50.0 to 63.0) | 0.503 |
| K-SCQ (Total) | 18.0 (13.0 to 20.0) | 16.0 (12.0 to 22.0) | 0.714 |
| K-VABS-2 |  |  |  |
| - Communication | 64.0 (57.0 to 70.0) | 66.0 (53.0 to 81.0) | 0.398 |
| - Daily living skills | 66.0 (59.0 to 69.0) | 66.0 (59.0 to 80.0) | 0.456 |
| - Socialization | 51.0 (46.5 to 54.0) | 50.0 (48.0 to 60.0) | 0.453 |
| - Motor | 67.0 (61.0 to 80.0) | 68.5 (58.0 to 80.0) | 0.919 |
| - Maladaptation | 18.0 (17.0 to 20.0) | 19.5 (19.0 to 20.0) | 0.333 |
| - Internalizing | 19.0 (17.5 to 21.0) | 20.5 (19.0 to 22.0) | 0.151 |
| - Externalizing | 17.0 (14.0 to 19.0) | 17.5 (14.0 to 19.0) | 0.869 |
| **Parents’ measures (for parents)**, median (IQR) |  |  |  |
| K-PSI-4-SF |  |  |  |
| - Total Stress scale | 103.0 (93.0 to 113.0) | 95.0 (88.0 to 109.0) | 0.278 |
| - Parental Distress (PD) | 34.0 (31.5 to 38.5) | 33.5 (28.0 to 40.0) | 0.743 |
| - Parent-Child Dysfunctional Interaction (P-CDI) | 34.0 (30.5 to 38.0) | 29.0 (26.0 to 38.0) | 0.165 |
| - Difficult Child (DC) | 32.5 (29.5 to 38.5) | 30.0 (26.0 to 37.0) | 0.427 |

*IQR: Interquartile range, K-CARS-2: Korean Childhood Autism Rating Scale-2, ADOS: Autism Diagnostic Observation Schedule, ABA: Applied Behavior Analysis, CGI-S: Clinical Global Impression-Severity of Illness, K-CBCL: Korean Child Behavior Checklist, K-SCQ: Korean versions of the Social Communication Questionnaire, K-VABS-2: Korean Vineland adaptive behavior scale-2, K-PSI-4: Korean version of Parenting Stress Index 4^th^ Edition-Short-Form*

**Table S2.** Demographic and baseline variable for intervention and control groups (a version of intention-to-treat).

|  | Group | | *P* |
| --- | --- | --- | --- |
| Characteristics | Intervention  (N=20) | Control  (N=25) |  |
| Sex, n (%) |  |  | 0.968 |
| - Male | 15 (75.0%) | 20 (80.0%) |  |
| - Female | 5 (25.0%) | 5 (20.0%) |  |
| Age (Months), median (IQR) | 49.0 (41.0 to 52.5) | 46.0 (42.0 to 58.0) | 0.672 |
| Severity, n (%) |  |  | 1.000 |
| - Mild | 4 (20.0%) | 5 (20.0%) |  |
| - Severe | 16 (80.0%) | 20 (80.0%) |  |
| Ethnicity, n (%) |  |  | 1.000 |
| - Asian | 20 (100.0%) | 25 (100.0%) |  |
| K-CARS-2, median (IQR) | 33.0 (32.0 to 36.2) | 34.5 (31.0 to 36.0) | 0.714 |
| ADOS module, n (%) |  |  | 0.423 |
| - Module 1 | 16 (80.0%) | 19 (76.0%) |  |
| - Module 2 | 3 (15.0%) | 6 (24.0%) |  |
| - Toddle | 1 (5.0%) | 0 (0.0%) |  |
| ADOS (Total), median (IQR) | 18.5 (17.0 to 20.0) | 16.0 (14.0 to 19.0) | 0.051 |
| - Social affect | 14.5 (12.5 to 16.0) | 12.0 (10.0 to 15.0) | 0.045 |
| - Repetitive behaviors | 4.0 (3.0 to 5.0) | 4.0 (2.0 to 5.0) | 0.522 |
| ADOS Comparison, n (%) |  |  | 0.661 |
| - Extreme | 11 (55.0%) | 11 (44.0%) |  |
| - Mild | 0 (0.0%) | 1 (4.0%) |  |
| - Moderate | 1 (5.0%) | 3 (12.0%) |  |
| - Moderate to Severe | 2 (10.0%) | 1 (4.0%) |  |
| - Severe | 6 (30.0%) | 9 (36.0%) |  |
| **Current Therapies, n (%)** |  |  |  |
| Ongoing intervention |  |  | 1.000 |
| - No | 3 (15.0%) | 4 (16.0%) |  |
| - Yes | 17 (85.0%) | 21 (84.0%) |  |
| ABA |  |  | 0.910 |
| - No | 14 (70.0%) | 19 (76.0%) |  |
| - Yes | 6 (30.0%) | 6 (24.0%) |  |
| Sensory integration therapy |  |  | 0.313 |
| - No | 5 (25.0%) | 11 (44.0%) |  |
| - Yes | 15 (75.0%) | 14 (56.0%) |  |
| Language therapy |  |  | 0.786 |
| - No | 4 (20.0%) | 7 (28.0%) |  |
| - Yes | 16 (80.0%) | 18 (72.0%) |  |
| Music therapy |  |  | 1.000 |
| - No | 20 (100.0%) | 24 (96.0%) |  |
| - Yes | 0 (0.0%) | 1 (4.0%) |  |
| Play therapy |  |  | 1.000 |
| - No | 12 (60.0%) | 14 (56.0%) |  |
| - Yes | 8 (40.0%) | 11 (44.0%) |  |
| Floortime |  |  | 0.571 |
| - No | 20 (100.0%) | 23 (92.0%) |  |
| - Yes | 0 (0.0%) | 2 (8.0%) |  |
| Cognition therapy |  |  | 1.000 |
| - No | 19 (95.0%) | 23 (92.0%) |  |
| - Yes | 1 (5.0%) | 2 (8.0%) |  |
| Occupational therapy |  |  | 0.883 |
| - No | 18 (90.0%) | 21 (84.0%) |  |
| - Yes | 2 (10.0%) | 4 (16.0%) |  |
| Art therapy |  |  | 0.91 |
| - No | 19 (95.0%) | 25 (100.0%) |  |
| - Yes | 1 (5.0%) | 0 (0.0%) |  |
| Developmental therapy |  |  | 1.000 |
| - No | 19 (95.0%) | 23 (92.0%) |  |
| - Yes | 1 (5.0%) | 2 (8.0%) |  |
| Social Skills therapy |  |  | 1.000 |
| - No | 19 (95.0%) | 23 (92.0%) |  |
| - Yes | 1 (5.0%) | 2 (8.0%) |  |
| Group therapy |  |  | 0.571 |
| - No | 20 (100.0%) | 23 (92.0%) |  |
| - Yes | 0 (0.0%) | 2 (8.0%) |  |
| **Physician measures** |  |  |  |
| CGI-S, median (IQR) | 3.0 (2.0 to 4.0) | 3.0 (2.0 to 4.0) | 0.944 |
| **Parents’ measures (for children)**, median (IQR) |  |  |  |
| K-CBCL (Total) | 57.5 (50.0 to 63.0) | 60.0 (55.0 to 64.0) | 0.529 |
| - Emotionally reactive | 50.0 (50.0 to 58.0) | 51.0 (50.0 to 63.0) | 0.108 |
| - Anxious/Depressed | 50.0 (50.0 to 58.0) | 50.0 (50.0 to 56.0) | 0.867 |
| - Somatic complaints | 54.0 (50.0 to 59.0) | 50.0 (50.0 to 54.0) | 0.507 |
| - Withdrawn | 66.5 (62.0 to 70.0) | 62.0 (58.0 to 65.0) | 0.107 |
| - Sleep problem | 50.0 (50.0 to 60.5) | 55.0 (50.0 to 62.0) | 0.531 |
| - Attention problems | 61.5 (55.0 to 66.0) | 59.0 (50.0 to 64.0) | 0.478 |
| - Aggressive behavior | 53.5 (50.0 to 57.5) | 57.0 (50.0 to 64.0) | 0.118 |
| - Other | 59.0 (55.0 to 64.5) | 64.0 (54.0 to 67.0) | 0.409 |
| - Internalizing domain | 56.5 (49.0 to 61.0) | 56.0 (52.0 to 60.0) | 0.982 |
| - Externalizing domain | 55.0 (49.0 to 63.0) | 59.0 (56.0 to 62.0) | 0.325 |
| - Affective disorder | 50.0 (50.0 to 58.0) | 50.0 (50.0 to 62.0) | 0.633 |
| - Anxiety disorder | 53.0 (50.0 to 59.0) | 53.0 (50.0 to 59.0) | 0.665 |
| - Pervasive developmental disorder | 69.0 (64.0 to 74.0) | 70.0 (66.0 to 76.0) | 0.429 |
| - Attention-Deficit/Hyperactivity disorder | 56.0 (52.0 to 64.5) | 59.0 (56.0 to 59.0) | 0.618 |
| - Oppositional defiant disorder | 53.5 (50.0 to 60.0) | 56.0 (50.0 to 63.0) | 0.376 |
| K-SCQ (Total) | 18.0 (13.0 to 20.0) | 16.0 (12.0 to 22.0) | 0.484 |
| K-VABS-2 |  |  |  |
| - Communication | 64.0 (57.0 to 70.0) | 67.0 (55.0 to 79.0) | 0.303 |
| - Daily living skills | 66.0 (59.0 to 69.0) | 68.0 (61.0 to 80.0) | 0.208 |
| - Socialization | 51.0 (46.5 to 54.0) | 50.0 (48.0 to 60.0) | 0.231 |
| - Motor | 67.0 (61.0 to 80.0) | 70.0 (58.0 to 80.0) | 1 |
| - Maladaptation | 18.0 (17.0 to 20.0) | 20.0 (19.0 to 20.0) | 0.224 |
| - Internalizing | 19.0 (17.5 to 21.0) | 21.0 (19.0 to 22.0) | 0.112 |
| - Externalizing | 17.0 (14.0 to 19.0) | 18.0 (14.0 to 19.0) | 0.579 |
| **Parents’ measures (for parents)**, median (IQR) |  |  |  |
| K-PSI-4-SF |  |  |  |
| - Total Stress scale | 103.0 (93.0 to 113.0) | 96.0 (90.0 to 111.0) | 0.437 |
| - Parental Distress (PD) | 34.0 (31.5 to 38.5) | 34.0 (32.0 to 40.0) | 0.982 |
| - Parent-Child Dysfunctional Interaction (P-CDI) | 34.0 (30.5 to 38.0) | 29.0 (27.0 to 38.0) | 0.163 |
| - Difficult Child (DC) | 32.5 (29.5 to 38.5) | 32.0 (28.0 to 38.0) | 0.591 |

*IQR: Interquartile range, K-CARS-2: Korean Childhood Autism Rating Scale-2, ADOS: Autism Diagnostic Observation Schedule, ABA: Applied Behavior Analysis, CGI-S: Clinical Global Impression-Severity of Illness, K-CBCL: Korean Child Behavior Checklist, K-SCQ: Korean versions of the Social Communication Questionnaire, K-VABS-2: Korean Vineland adaptive behavior scale-2, K-PSI-4: Korean version of Parenting Stress Index 4^th^ Edition-Short-Form*

**Table S3.** Comparison between outcome variables for the intervention and control groups (a version of per protocol).

| Characteristics | Intervention | | | | Control | | | | Median Difference  (Median, 95% CI) | *P* |
| --- | --- | --- | --- | --- | --- | --- | --- | --- | --- | --- |
|  | Pre  (N=20)  (Median, IQR) | Post  (N=20)  (Median, IQR) | Median Difference  (Median, 95% CI) | *P* | Pre  (N=22)  (Median, IQR) | Post  (N=22)  (Median, IQR) | Median Difference  (Median, 95% CI) | *P* |  |  |
| **Primary Outcomes** |  |  |  |  |  |  |  |  |  |  |
| K-CBCL (Total)^a^ | 57.5 (50.0 to 63.0) | 58.0 (50.0 to 63.0) | -0.5  (-4.5 to 3.0) | 0.9106 | 60.5 (55.0 to 64.0) | 55.5 (52.0 to 65.0) | -2.0  (-6.0 to 2.0) | 0.4312 | 2.0  (-4.0 to 6.0) | 0.4955 |
| - Emotionally reactive | 50.0 (50.0 to 58.0) | 50.0 (50.0 to 61.5) | 3.0  (-4.0 to 6.5) | 0.3479 | 53.0 (50.0 to 63.0) | 56.5 (50.0 to 60.0) | 0.5  (-2.5 to 4.0) | 0.7362 | 0.0  (-2.0 to 5.0) | 0.7864 |
| - Anxious/Depressed | 50.0 (50.0 to 58.0) | 50.0 (50.0 to 58.0) | 0.5  (-7.0 to 8.0) | 0.8937 | 50.0 (50.0 to 56.0) | 50.0 (50.0 to 56.0) | 0.0  (-5.5 to 6.0) | 1.0000 | 0.0  (-4.0 to 2.0) | 0.7287 |
| - Somatic complaints | 54.0 (50.0 to 59.0) | 52.0 (50.0 to 59.0) | 0.3  (-9.5 to 4.5) | 0.9370 | 50.0 (50.0 to 54.0) | 50.0 (50.0 to 50.0) | -4.0  (-11.0 to 2.5) | 0.1366 | 0.0  (0.0 to 4.0) | 0.2358 |
| - Withdrawn | 66.5 (62.0 to 70.0) | 68.0 (60.0 to 71.5) | 1.0  (-4.5 to 6.5) | 0.7761 | 62.0 (58.0 to 65.0) | 68.0 (62.0 to 70.0) | 4.0  (-2.5 to 8.0) | 0.2267 | -2.0  (-8.0 to 4.0) | 0.5024 |
| - Sleep problem | 50.0 (50.0 to 60.5) | 50.0 (50.0 to 59.0) | -1.0  (-9.0 to 6.0) | 0.6589 | 55.0 (50.0 to 62.0) | 52.5 (50.0 to 59.0) | -4.5  (-8.5 to 1.5) | 0.1071 | 0.0  (-2.0 to 6.0) | 0.4622 |
| - Attention problems | 61.5 (55.0 to 66.0) | 61.5 (55.0 to 66.0) | 0.0  (-4.5 to 6.5) | 0.9497 | 59.0 (50.0 to 64.0) | 61.5 (55.0 to 68.0) | 1.5  (-2.0 to 7.0) | 0.3953 | -1.0  (-6.0 to 4.0) | 0.5511 |
| - Aggressive behavior | 53.5 (50.0 to 57.5) | 54.5 (50.0 to 61.5) | 2.5  (-1.5 to 6.5) | 0.2433 | 57.0 (50.0 to 64.0) | 53.0 (50.0 to 58.0) | -3.0  (-7.0 to 1.5) | 0.2043 | 3.0  (0.0 to 7.0) | 0.0652 |
| - Other | 59.0 (55.0 to 64.5) | 60.0 (52.0 to 63.0) | -1.5  (-5.0 to 2.5) | 0.4201 | 64.0 (54.0 to 67.0) | 58.0 (52.0 to 68.0) | -2.5  (-7.5 to 2.5) | 0.2852 | 1.0  (-4.0 to 6.0) | 0.5112 |
| - Internalizing domain | 56.5 (49.0 to 61.0) | 55.0 (49.0 to 63.5) | -0.2  (-4.0 to 3.5) | 0.8718 | 56.0 (54.0 to 60.0) | 54.0 (50.0 to 62.0) | -0.5  (-4.5 to 4.5) | 0.8309 | 0.0  (-4.0 to 4.0) | 0.9597 |
| - Externalizing domain | 55.0 (49.0 to 63.0) | 57.0 (49.5 to 63.0) | 1.0  (-3.0 to 4.0) | 0.5488 | 58.5 (51.0 to 62.0) | 54.0 (51.0 to 62.0) | -1.5  (-4.5 to 2.5) | 0.4948 | 2.0  (-3.0 to 7.0) | 0.4123 |
| - Affective disorder | 50.0 (50.0 to 58.0) | 52.5 (50.0 to 58.0) | 0.0  (-8.0 to 8.0) | 1.0000 | 50.0 (50.0 to 62.0) | 50.0 (50.0 to 62.0) | -1.5  (-8.5 to 6.5) | 0.8582 | 0.0  (0.0 to 4.0) | 0.4295 |
| - Anxiety disorder | 53.0 (50.0 to 59.0) | 50.0 (50.0 to 59.0) | -1.5  (-6.0 to 7.0) | 0.6987 | 53.0 (50.0 to 59.0) | 50.0 (50.0 to 56.0) | -0.9  (-7.5 to 5.5) | 0.7339 | 0.0  (-3.0 to 3.0) | 0.7436 |
| - Pervasive developmental disorder | 69.0 (64.0 to 74.0) | 69.5 (59.5 to 72.0) | -2.1  (-8.5 to 2.5) | 0.4073 | 69.5 (66.0 to 76.0) | 69.5 (66.0 to 76.0) | 1.0  (-4.5 to 4.0) | 0.7361 | -1.0  (-5.0 to 2.0) | 0.4090 |
| - Attention-Deficit/Hyperactivity disorder | 56.0 (52.0 to 64.5) | 56.0 (52.0 to 63.0) | 1.0  (-4.0 to 7.5) | 0.3728 | 59.0 (56.0 to 59.0) | 59.0 (56.0 to 63.0) | 2.0  (-2.5 to 7.0) | 0.3480 | 0.0  (-5.0 to 4.0) | 0.9898 |
| - Oppositional defiant disorder | 53.5 (50.0 to 60.0) | 53.5 (50.0 to 60.0) | 1.5  (-3.5 to 7.0) | 0.5317 | 56.0 (50.0 to 63.0) | 51.0 (51.0 to 63.0) | -1.8  (-8.0 to 4.0) | 0.6408 | 0.0  (-2.0 to 5.0) | 0.8770 |
| **Physician measures** |  |  |  |  |  |  |  |  |  |  |
| CGI-S^a^ | 3.0 (2.0 to 4.0) | 3.0 (2.0 to 3.0) | -0.5  (-2.0 to 1.0) | 0.4007 | 3.0 (2.0 to 4.0) | 3.0 (2.0 to 3.0) | -1.0  (-2.0 to 1.0) | 0.2274 | 0.0  (0.0 to 0.5) | 0.6953 |
| **Secondary Outcomes** |  |  |  |  |  |  |  |  |  |  |
| K-SCQ (Total)^a^ | 18.0 (13.0 to 20.0) | 15.0 (9.5 to 20.0) | -2.0  (-4.0 to 1.0) | 0.1966 | 16.0 (12.0 to 22.0) | 16.5 (9.0 to 20.0) | -1.5  (-3.0 to 0.0) | 0.0787 | 0.0  (-3.0 to 3.0) | 0.8995 |
| K-VABS-2^b^ |  |  |  |  |  |  |  |  |  |  |
| - Communication | 64.0 (57.0 to 70.0) | 68.0 (56.0 to 75.0) | 4.0  (0.0 to 8.0) | 0.0339 | 66.0 (53.0 to 81.0) | 73.0 (52.0 to 85.0) | 0.0  (-5.0 to 3.0) | 0.8365 | 4.0  (0.0 to 10.0) | 0.0904 |
| - Daily living skills | 66.0 (59.0 to 69.0) | 69.0 (63.0 to 74.0) | 4.5  (-2.5 to 14.5) | 0.1239 | 66.0 (59.0 to 80.0) | 66.0 (49.0 to 76.0) | -5.1  (-9.5 to 0.5) | 0.0584 | 6.0  (1.0 to 13.0) | 0.0173 |
| - Socialization | 51.0 (46.5 to 54.0) | 55.0 (48.0 to 62.5) | 7.0  (2.5 to 12.0) | 0.0032 | 50.0 (48.0 to 60.0) | 51.0 (48.0 to 56.0) | -1.5  (-5.5 to 2.0) | 0.5522 | 7.0  (2.0 to 13.0) | 0.0084 |
| - Motor | 67.0 (61.0 to 80.0) | 71.5 (62.5 to 83.5) | 3.4  (-2.0 to 10.0) | 0.1773 | 68.5 (58.0 to 80.0) | 74.5 (64.0 to 83.0) | 4.0  (-1.0 to 8.0) | 0.1022 | 0.0  (-6.0 to 5.0) | 0.7809 |
| - Maladaptation | 18.0 (17.0 to 20.0) | 18.5 (17.5 to 20.5) | 0.5  (-1.0 to 2.0) | 0.5072 | 19.5 (19.0 to 20.0) | 19.5 (17.0 to 20.0) | -1.0  (-3.0 to 1.0) | 0.1803 | 1.0  (-1.0 to 2.0) | 0.2157 |
| - Internalizing | 19.0 (17.5 to 21.0) | 18.0 (16.5 to 21.5) | -0.5  (-2.5 to 1.5) | 0.5403 | 20.5 (19.0 to 22.0) | 21.0 (18.0 to 22.0) | -1.0  (-2.5 to 1.5) | 0.7736 | 0.0  (-2.0 to 2.0) | 0.8590 |
| - Externalizing | 17.0 (14.0 to 19.0) | 16.5 (13.5 to 19.5) | 0.5  (-2.0 to 2.5) | 0.7824 | 17.5 (14.0 to 19.0) | 16.5 (13.0 to 19.0) | -0.7  (-2.5 to 1.0) | 0.3824 | 1.0  (-1.0 to 2.0) | 0.3454 |
| **Parents’ measures**  **(for parents)** |  |  |  |  |  |  |  |  |  |  |
| K-PSI-4-SF^a^ |  |  |  |  |  |  |  |  |  |  |
| Total Stress scale | 103.0 (93.0 to 113.0) | 106.5 (93.0 to 116.0) | 0.5  (-4.5 to 6.5) | 0.8788 | 95.0 (88.0 to 109.0) | 93.5 (87.0 to 108.0) | -3.5  (-9.0 to 3.0) | 0.2041 | 4.0  (-3.0 to 11.0) | 0.1855 |
| Parental Distress (PD) | 34.0 (31.5 to 38.5) | 37.0 (32.0 to 41.5) | 1.5  (-1.5 to 5.0) | 0.2944 | 33.5 (28.0 to 40.0) | 32.5 (28.0 to 38.0) | -1.5  (-3.5 to 0.5) | 0.1489 | 2.0  (0.0 to 6.0) | 0.0670 |
| Parent-Child Dysfunctional Interaction (P-CDI) | 34.0 (30.5 to 38.0) | 33.5 (29.0 to 38.0) | -0.5  (-2.5 to 2.0) | 0.6774 | 29.0 (26.0 to 38.0) | 30.0 (28.0 to 34.0) | -1.5  (-4.0 to 1.5) | 0.3643 | 1.0  (-2.0 to 4.0) | 0.7234 |
| Difficult Child (DC) | 32.5 (29.5 to 38.5) | 33.5 (28.5 to 36.5) | 0.0  (-2.5 to 2.5) | 0.8493 | 30.0 (26.0 to 37.0) | 30.5 (28.0 to 37.0) | -0.5  (-3.0 to 1.5) | 0.5359 | 0.5  (-3.0 to 3.0) | 0.7714 |

*^a^ Lower score indicate increased ability*

*^b^ Higher score indicate increased ability*

*IQR: Interquartile range, 95% CI: 95% Confidence Interval, K-CARS-2: Korean Childhood Autism Rating Scale-2, ADOS: Autism Diagnostic Observation Schedule, ABA: Applied Behavior Analysis, CGI-S: Clinical Global Impression-Severity of Illness, K-CBCL: Korean Child Behavior Checklist, K-SCQ: Korean versions of the Social Communication Questionnaire, K-VABS-2: Korean Vineland adaptive behavior scale-2, K-PSI-4: Korean version of Parenting Stress Index 4th Edition*

**Table S4.** Comparison between outcome variables for the intervention and control groups (a version of Intention-to-treat).

| Characteristics | Intervention | | | | Control | | | | Median Difference  (Median, 95% CI) | *P* |
| --- | --- | --- | --- | --- | --- | --- | --- | --- | --- | --- |
|  | Pre  (N=20)  (Median, IQR) | Post  (N=20)  (Median, IQR) | Median Difference  (Median, 95% CI) | *P* | Pre  (N=25)  (Median, IQR) | Post  (N=25)  (Median, IQR) | Median Difference  (Median, 95% CI) | *P* |  |  |
| **Primary Outcomes** |  |  |  |  |  |  |  |  |  |  |
| K-CBCL (Total)^a^ | 57.5 (50.0 to 63.0) | 58.0 (50.0 to 63.0) | -0.5  (-4.5 to 3.0) | 0.9106 | 60.0  (55.0 to 64.0) | 55.0 (52.0 to 65.0) | -3.00  (-6.0 to 1.50) | 0.1987 | 2.00  (-3.00 to 7.00)) | 0.3364 |
| - Emotionally reactive | 50.0 (50.0 to 58.0) | 50.0 (50.0 to 61.5) | 3.0  (-4.0 to 6.5) | 0.3479 | 51.0 (50.0 to 63.0) | 55.0 (50.0 to 60.0) | 0.50  (-2.50 to 4.00) | 0.7758 | 0.00  (-1.00 to 4.00) | 0.6298 |
| - Anxious/Depressed | 50.0 (50.0 to 58.0) | 50.0 (50.0 to 58.0) | 0.5  (-7.0 to 8.0) | 0.8937 | 50.0 (50.0 to 56.0) | 50.0 (50.0 to 56.0) | -0.13  (-6.00 to 4.00) | 0.8749 | 0.00  (-2.00 to 2.00) | 0.8079 |
| - Somatic complaints | 54.0 (50.0 to 59.0) | 52.0 (50.0 to 59.0) | 0.3  (-9.5 to 4.5) | 0.9370 | 50.0 (50.0 to 54.0) | 50.0 (50.0 to 54.0) | -4.00  (-11.00 to 2.50) | 0.0943 | 0.00  (0.00 to 4.00) | 0.1924 |
| - Withdrawn | 66.5 (62.0 to 70.0) | 68.0 (60.0 to 71.5) | 1.0  (-4.5 to 6.5) | 0.7761 | 62.0 (58.0 to 65.0) | 68.0 (58.0 to 70.0) | 2.00  (-3.50 to 7.50) | 0.4433 | 0.00  (-7.00 to 5.00) | 0.8097 |
| - Sleep problem | 50.0 (50.0 to 60.5) | 50.0 (50.0 to 59.0) | -1.0  (-9.0 to 6.0) | 0.6589 | 55.0 (50.0 to 62.0) | 50.0 (50.0 to 59.0) | -4.00  (-8.00 to 1.50) | 0.1456 | 0.00  (-3.00 to 5.00) | 0.5275 |
| - Attention problems | 61.5 (55.0 to 66.0) | 61.5 (55.0 to 66.0) | 0.0  (-4.5 to 6.5) | 0.9497 | 59.0 (50.0 to 64.0) | 64.0 (55.0 to 68.0) | 1.50  (-2.50 to 7.00) | 0.4438 | -0.18  (-5.00 to 4.00) | 0.6707 |
| - Aggressive behavior | 53.5 (50.0 to 57.5) | 54.5 (50.0 to 61.5) | 2.5  (-1.5 to 6.5) | 0.2433 | 57.0 (50.0 to 64.0) | 54.0 (50.0 to 58.0) | -3.50  (-7.00 to 0.50) | 0.0796 | 3.00  (0.00 to 8.00) | 0.0279 |
| - Other | 59.0 (55.0 to 64.5) | 60.0 (52.0 to 63.0) | -1.5  (-5.0 to 2.5) | 0.4201 | 64.0 (54.0 to 67.0) | 57.0 (52.0 to 68.0) | -3.00  (-7.00 to 1.50) | 0.1533 | 2.00  (-3.00 to 6.00) | 0.3777 |
| - Internalizing domain | 56.5 (49.0 to 61.0) | 55.0 (49.0 to 63.5) | -0.2  (-4.0 to 3.5) | 0.8718 | 56.0 (52.0 to 60.0) | 54.0 (50.0 to 62.0) | -1.50  (-5.00 to 3.50) | 0.5451 | 0.66  (-4.00 to 5.00) | 0.8546 |
| - Externalizing domain | 55.0 (49.0 to 63.0) | 57.0 (49.5 to 63.0) | 1.0  (-3.0 to 4.0) | 0.5488 | 59.0 (56.0 to 62.0) | 54.0 (51.0 to 60.0) | -2.00  (-5.00 to 1.50) | 0.2204 | 3.00  (-2.00 to 8.00) | 0.2341 |
| - Affective disorder | 50.0 (50.0 to 58.0) | 52.5 (50.0 to 58.0) | 0.0  (-8.0 to 8.0) | 1.0000 | 50.0 (50.0 to 62.0) | 50.0 (50.0 to 62.0) | -2.00  (-8.50 to 6.50) | 0.6452 | 0.00  (0.00 to 4.00) | 0.3456 |
| - Anxiety disorder | 53.0 (50.0 to 59.0) | 50.0 (50.0 to 59.0) | -1.5  (-6.0 to 7.0) | 0.6987 | 53.0 (50.0 to 59.0) | 50.0 (50.0 to 56.0) | -0.27  (-7.50 to 6.00) | 0.9438 | 0.00  (-3.00 to 0.00) | 0.6020 |
| - Pervasive developmental disorder | 69.0 (64.0 to 74.0) | 69.5 (59.5 to 72.0) | -2.1  (-8.5 to 2.5) | 0.4073 | 70.0 (66.0 to 76.0) | 69.0 (64.0 to 76.0) | -1.50  (-6.00 to 2.50) | 0.5725 | 0.00  (-4.00 to 3.00) | 0.7825 |
| - Attention-Deficit/Hyperactivity disorder | 56.0 (52.0 to 64.5) | 56.0 (52.0 to 63.0) | 1.0  (-4.0 to 7.5) | 0.3728 | 59.0 (56.0 to 59.0) | 59.0 (56.0 to 63.0) | 1.50  (-2.00 to 6.50) | 0.3400 | 0.00  (-4.00 to 4.00) | 0.9908 |
| - Oppositional defiant disorder | 53.5 (50.0 to 60.0) | 53.5 (50.0 to 60.0) | 1.5  (-3.5 to 7.0) | 0.5317 | 56.0 (50.0 to 63.0) | 51.0 (51.0 to 63.0) | -2.45  (-8.00 to 2.00) | 0.2764 | 1.00  (-1.00 to 6.00) | 0.4631 |
| **Physician measures** |  |  |  |  |  |  |  |  |  |  |
| CGI-S^a^ | 3.0 (2.0 to 4.0) | 3.0 (2.0 to 3.0) | -0.5  (-2.0 to 1.0) | 0.4007 | 3.0 (2.0 to 4.0) | 3.0 (2.0 to 3.0) | -1.00  (-2.00 to 0.25) | 0.2526 | 0.00  (0.00 to 0.50) | 0.7723 |
| **Secondary Outcomes** |  |  |  |  |  |  |  |  |  |  |
| K-SCQ (Total)^a^ | 18.0 (13.0 to 20.0) | 15.0 (9.5 to 20.0) | -2.0  (-4.0 to 1.0) | 0.1966 | 16.0 (12.0 to 22.0) | 15.0 (8.0 to 19.0) | -1.50  (-3.50 to 1.00) | 0.0470 | 0.00  (0.00 to 0.50) | 0.9726 |
| K-VABS-2^b^ |  |  |  |  |  |  |  |  |  |  |
| - Communication | 64.0 (57.0 to 70.0) | 68.0 (56.0 to 75.0) | 4.0  (0.0 to 8.0) | 0.0339 | 67.0 (55.0 to 79.0) | 73.0 (55.0 to 83.0) | 0.00  (-4.00 to 3.00) | 0.9090 | 4.00  (0.00 to 10.00) | 0.0855 |
| - Daily living skills | 66.0 (59.0 to 69.0) | 69.0 (63.0 to 74.0) | 4.5  (-2.5 to 14.5) | 0.1239 | 68.0 (61.0 to 80.0) | 70.0 (54.0 to 80.0) | -3.00  (-8.00 to 2.00) | 0.1303 | 5.44  (0.00 to 11.00) | 0.0444 |
| - Socialization | 51.0 (46.5 to 54.0) | 55.0 (48.0 to 62.5) | 7.0  (2.5 to 12.0) | 0.0032 | 50.0 (48.0 to 60.0) | 52.0 (48.0 to 58.0) | -2.00  (-5.50 to 2.00) | 0.3420 | 7.00  (2.00 to 12.00) | 0.0038 |
| - Motor | 67.0 (61.0 to 80.0) | 71.5 (62.5 to 83.5) | 3.4  (-2.0 to 10.0) | 0.1773 | 70.0 (58.0 to 80.0) | 76.0 (64.0 to 87.0) | 4.50  (0.00 to 8.50) | 0.0374 | -1.00  (-6.00 to 4.00) | 0.6222 |
| - Maladaptation | 18.0 (17.0 to 20.0) | 18.5 (17.5 to 20.5) | 0.5  (-1.0 to 2.0) | 0.5072 | 20.0 (19.0 to 20.0) | 19.0 (17.0 to 20.0) | -1.00  (-2.50 to 0.50) | 0.1083 | 1.00  (0.00 to 3.00) | 0.1765 |
| - Internalizing | 19.0 (17.5 to 21.0) | 18.0 (16.5 to 21.5) | -0.5  (-2.5 to 1.5) | 0.5403 | 21.0 (19.0 to 22.0) | 20.0 (16.0 to 21.0) | -1.00  (-3.00 to 0.50) | 0.2646 | 0.00  (-2.00 to 2.00) | 0.8271 |
| - Externalizing | 17.0 (14.0 to 19.0) | 16.5 (13.5 to 19.5) | 0.5  (-2.0 to 2.5) | 0.7824 | 18.0 (14.0 to 19.0) | 17.0 (14.0 to 19.0) | -0.50  (-2.50 to 1.00) | 0.4127 | 0.00  (-1.00 to 2.00) | 0.3733 |
| **Parents’ measures**  **(for parents)** |  |  |  |  |  |  |  |  |  |  |
| K-PSI-4-SF^a^ |  |  |  |  |  |  |  |  |  |  |
| Total Stress scale | 103.0 (93.0 to 113.0) | 106.5 (93.0 to 116.0) | 0.5  (-4.5 to 6.5) | 0.8788 | 96.0 (90.0 to 111.0) | 93.0 (87.0 to 106.0) | -6.00  (-11.00 to 0.50) | 0.0592 | 5.00  (0.00 to 13.00) | 0.0608 |
| Parental Distress (PD) | 34.0 (31.5 to 38.5) | 37.0 (32.0 to 41.5) | 1.5  (-1.5 to 5.0) | 0.2944 | 34.0 (32.0 to 40.0) | 33.0 (29.0 to 38.0) | -2.00  (-3.50 to 0.00) | 0.0669 | 3.00  (0.00 to 6.00) | 0.0370 |
| Parent-Child Dysfunctional Interaction (P-CDI) | 34.0 (30.5 to 38.0) | 33.5 (29.0 to 38.0) | -0.5  (-2.5 to 2.0) | 0.6774 | 29.0 (27.0 to 38.0) | 30.0 (27.0 to 34.0) | -2.00  (-4.50 to 1.50) | 0.2166 | 1.00  (-2.00 to 5.00) | 0.5823 |
| Difficult Child (DC) | 32.5 (29.5 to 38.5) | 33.5 (28.5 to 36.5) | 0.0  (-2.5 to 2.5) | 0.8493 | 32.0 (28.0 to 38.0) | 31.0 (28.0 to 37.0) | -1.50  (-3.50 to 1.00) | 0.1954 | 1.00  (-2.00 to 4.00) | 0.4031 |

*^a^ Lower score indicate increased ability*

*^b^ Higher score indicate increased ability*

*IQR: Interquartile range, 95% CI: 95% Confidence Interval, K-CARS-2: Korean Childhood Autism Rating Scale-2, ADOS: Autism Diagnostic Observation Schedule, ABA: Applied Behavior Analysis, CGI-S: Clinical Global Impression-Severity of Illness, K-CBCL: Korean Child Behavior Checklist, K-SCQ: Korean versions of the Social Communication Questionnaire, K-VABS-2: Korean Vineland adaptive behavior scale-2, K-PSI-4: Korean version of Parenting Stress Index 4th Edition*

**Table S5.** Difference in pre and post evaluation values by session completion rate.

| Characteristics | Session completion rate | | | | |
| --- | --- | --- | --- | --- | --- |
|  | 100%^1^  (N=5)  Median Difference  (Median, 95% CI) | 90%^2^  (N=6)  Median Difference  (Median, 95% CI) | 80%^3^  (N=7)  Median Difference  (Median, 95% CI) | 60%^4^  (N=8)  Median Difference  (Median, 95% CI) | 40%^5^ (N=9)  Median Difference  (Median, 95% CI) |
| **Primary Outcomes** |  |  |  |  |  |
| K-CBCL (Total)^a^ | -3.5  (-12.0 to 5.0) | -3.0  (-8.5 to 4.5) | -3.4  (-8.5 to 4.5) | -3.5  (-8.5 to 4.5) | -3.5  (-8.5 to 3.0) |
| - Emotionally reactive | 0.5  (0.5 to 0.5) | 2.0  (-1.0 to 5.0) | -0.2  (-9.0 to 5.0) | -1.0  (-9.0 to 5.0) | -1.0  (-9.0 to 5.0) |
| - Anxious/Depressed | 5.5  (5.5 to 5.5) | 6.8  (-2.0 to 13.0) | 6.8  (-2.0 to 13.0) | 6.8  (-2.0 to 13.0) | 3.1  (-6.0 to 13.0) |
| - Somatic complaints | -2.0  (-2.0 to -2.0) | -2.0  (-2.0 to -2.0) | -0.8  (-9.0 to 5.0) | -0.8  (-9.0 to 5.0) | 4.4  (-2.0 to 5.0) |
| - Withdrawn | -4.5  (-14.0 to 5.0) | -2.0  (-8.0 to 5.0) | -2.0  (-8.0 to 5.0) | -3.5  (-10.5 to 5.0) | -3.1  (-11.0 to 6.0) |
| - Sleep problem | -12.0  (NA to NA) | -8.5  (-8.5 to -8.5) | -9.1  (-12.0 to -12.0) | -9.1  (-12.0 to -12.0) | -8.3  (-12.0 to -1.5) |
| - Attention problems | 0.0  (-4.0 to 4.0) | 2.5  (-4.0 to 9.0) | 0.0  (-7.5 to 9.0) | 0.0  (-7.5 to 9.0) | 0.0  (-7.5 to 9.0) |
| - Aggressive behavior | -1.0  (-1.0 to -1.0) | -1.0  (-5.0 to 3.0) | -0.4  (-5.0 to 3.0) | 0.5  (-5.0 to 3.0) | -0.5  (-3.0 to 2.5) |
| - Other | 0.5  (-7.0 to 9.0) | 0.0  (-6.5 to 7.0) | -2.1  (-6.5 to 3.5) | -4.4  (-7.0 to 1.5) | -4.5  (-7.0 to 2.5) |
| - Internalizing domain | -2.0  (-7.0 to 2.0) | -1.0  (-7.0 to 5.0) | -1.3  (-8.0 to 5.5) | -2.0  (-8.0 to 4.0) | -2.0  (-7.0 to 2.0) |
| - Externalizing domain | -3.0  (-8.0 to 3.0) | -2.0  (-7.0 to 3.0) | -2.0  (-5.0 to 3.0) | -0.9  (-7.0 to 3.5) | -1.0  (-7.0 to 3.0) |
| - Affective disorder | -8.0  (NA to NA) | -2.5  (-2.5 to -2.5) | -3.4  (-8.0 to -8.0) | -3.4  (-8.0 to -8.0) | -8.0  (-8.0 to -8.0) |
| - Anxiety disorder | 1.2  (-9.0 to 17.0) | 1.2  (-9.0 to 17.0) | 0.9  (-9.0 to 17.0) | 0.9  (-9.0 to 17.0) | 3.0  (-9.0 to 17.0) |
| - Pervasive developmental disorder | -6.5  (-12.0 to 3.0) | -6.5  (-12.0 to 3.0) | -6.0  (-10.0 to -0.5) | -6.5  (-12.5 to -0.5) | -6.5  (-12.5 to -0.5) |
| - Attention-Deficit/Hyperactivity disorder | -0.8  (-4.0 to 4.0) | 0.4  (-2.0 to 4.0) | -1.5  (-4.5 to 4.0) | -1.5  (-4.5 to 4.0) | -1.5  (-5.5 to 4.0) |
| - Oppositional defiant disorder | -1.0  (NA to NA) | -1.0  (NA to NA) | -1.0  (NA to NA) | -0.2  (-1.0 to -1.0) | 0.8  (-1.0 to 1.0) |
| **Physician measures** |  |  |  |  |  |
| CGI-S^a^ | -0.5  (-0.5 to -0.5) | -0.2  (1.0 to 1.0) | -0.4  (-0.5 to 1.0) | -0.4  (-0.5 to 1.0) | -0.4  (-0.5 to 1.0) |
| **Secondary Outcomes** |  |  |  |  |  |
| K-SCQ (Total)^a^ | -5.5  (-7.0 to -4.0) | -5.0  (-6.0 to -1.0) | -4.5  (-6.0 to -0.5) | -4.0  (-6.5 to -0.5) | -3.0  (-6.0 to 1.0) |
| K-VABS-2^b^ |  |  |  |  |  |
| - Communication | 8.0  (-8.0 to 24.0) | 8.0  (-2.0 to 16.0) | 3.8  (-6.0 to 17.0) | 3.0  (-5.0 to 16.0) | 3.0  (-4.0 to 11.0) |
| - Daily living skills | 1.0  (-15.0 to 6.0) | 1.0  (-6.5 to 5.0) | 1.0  (-6.5 to 5.0) | 1.0  (-6.5 to 5.0) | 2.5  (-6.5 to 12.5) |
| - Socialization | 4.8  (-4.0 to 15.0) | 5.5  (-4.0 to 15.0) | 5.5  (1.0 to 10.5) | 5.5  (0.0 to 10.5) | 5.8  (0.0 to 12.0) |
| - Motor | 10.8  (2.0 to 22.0) | 10.8  (2.0 to 22.0) | 8.0  (2.0 to 22.0) | 5.0  (0.0 to 13.0) | 5.5  (-0.5 to 17.5) |
| - Maladaptation | 0.5  (-3.0 to 5.0) | 0.5  (-3.0 to 5.0) | 0.5  (-3.0 to 5.0) | -0.5  (-2.0 to 2.0) | -1.0  (-2.0 to 2.0) |
| - Internalizing | -1.5  (-2.0 to -0.5) | -0.5  (-2.0 to 1.0) | -1.0  (-3.0 to 1.0) | -1.5  (-3.0 to 1.0) | -1.5  (-3.0 to 1.0) |
| - Externalizing | -1.2  (-4.0 to 3.0) | -1.3  (-4.0 to 3.0) | -1.3  (-4.0 to 3.0) | -0.5  (-2.5 to 3.0) | -0.5  (-2.5 to 3.0) |
| **Parents’ measures**  **(for parents)** |  |  |  |  |  |
| K-PSI-4-SF^a^ |  |  |  |  |  |
| Total Stress scale | 9.5  (1.5 to 17.0) | 6.5  (-4.0 to 17.0) | 4.1  (-8.5 to 20.0) | 2.4  (-7.5 to 15.0) | 1.5  (-6.5 to 12.0) |
| Parental Distress (PD) | 5.5  (-2.0 to 14.0) | 5.0  (-1.5 to 9.5) | 1.5  (-7.0 to 13.0) | 2.0  (-6.5 to 10.0) | 2.5  (-3.0 to 9.5) |
| Parent-Child Dysfunctional Interaction (P-CDI) | 1.3  (-6.0 to 3.0) | -1.5  (-6.0 to 2.0) | -1.5  (-4.0 to 2.0) | -2.0  (-5.5 to 2.0) | -1.7  (-5.5 to 2.0) |
| Difficult Child (DC) | 4.6  (-2.0 to 10.0) | 4.0  (0.5 to 8.0) | 3.0  (0.5 to 8.0) | 2.9  (-5.0 to 9.0) | 1.3  (-5.0 to 6.5) |

*^1^ Groups with a session completion rate of 100% for the program*

*^2^ Groups with a session completion rate of 90% or higher for the program*

*^3^ Groups with a session completion rate of 80% or higher for the program*

*^4^ Groups with a session completion rate of 60% or higher for the program*

*^5^ Groups with a session completion rate of 40% or higher for the program*

*^a^ Lower score indicate increased ability*

*^b^ Higher score indicate increased ability*

*95% CI: 95% Confidence Interval, K-CARS-2: Korean Childhood Autism Rating Scale-2, ADOS: Autism Diagnostic Observation Schedule, ABA: Applied Behavior Analysis, CGI-S: Clinical Global Impression-Severity of Illness, K-CBCL: Korean Child Behavior Checklist, K-SCQ: Korean versions of the Social Communication Questionnaire, K-VABS-2: Korean Vineland adaptive behavior scale-2, K-PSI-4-SF: Korean Parenting Stress Index 4th Edition – Short Form*

**Figure S1.** Comparison between CGI-I for the intervention and control groups.

*CGI-I: Clinical Global Impression-Improvement*

**Figure S2.** Correlate differences in pre- and postintervention evaluation results by session completion rate.
